# S100A4 promotes lung tumor development through β-catenin pathway-mediated autophagy inhibition

**DOI:** 10.1038/s41419-018-0319-1

**Published:** 2018-02-15

**Authors:** Shasha Hou, Tian Tian, Dianwen Qi, Kaiji Sun, Qi Yuan, Ziling Wang, Zhihai Qin, Zhenlong Wu, Zhinan Chen, Jinhua Zhang

**Affiliations:** 1 College of Life Science and Bioengineering, Beijing Jiaotong University, Beijing, 100044 China; 2 Department of Musculoskeletal Tumor, Hebei Medical University Third Hospital, No.139 Ziqiang Road, Shijiazhuang, Hebei 050051 China; 3 State Key Laboratory of Animal Nutrition, College of Animal Science and Technology, China Agricultural University, Beijing, 100193 China; 4 Key Laboratory of Protein and Peptide Pharmaceuticals, CAS Center for Excellence in Biomacromolecules, Institute of Biophysics, Chinese Academy of Sciences, Beijing, 100101 China; 5 Cell Engineering Research Center and Department of Cell Biology, State Key Laboratory of Cancer, Fourth Military Medical University, Xi’an, China

## Abstract

Autophagy has emerged as a critical pathway in tumor development. S100A4 plays important roles in tumor metastasis, but its role in regulating autophagy has not been well characterized. In this study, we found that S100A4 was significantly upregulated in lung adenocarcinoma tissues. Clinical investigation demonstrated that high expression level of S100A4 was associated with tumor size and advanced tumor grades of lung adenocarcinoma patients. Moreover, our results revealed that extracellular S100A4 or overexpression of S100A4 inhibited starvation-induced autophagy and promoted cell proliferation in lung cancer cells in vitro; whereas small interfering RNA (siRNA)-mediated suppression of S100A4 increased autophagy and reduced cell viability in both A549 and LLC cells. Additionally, S100A4 inhibited starvation-induced autophagy to promote tumor cell viability via the Wnt pathway. Increased expression of β-catenin consistently led to a decreased LC3-II protein abundance. Further, the inhibitory effect of S100A4 on autophagy and its promotion role in cell proliferation was abolished in A549 and LLC cells using the receptor for advanced glycation end products (RAGE)-specific inhibitor (FPS-ZM1). S100A4-deficient mice showed retarded tumor development. This effect was well correlated with increased expression of autophagy markers. Our findings demonstrate that S100A4 promotes lung tumor development through inhibiting autophagy in a β-catenin signaling and S100A4 receptor RAGE-dependent manner, which provides a novel mechanism of S100A4-associated promotion of tumor development.

## Introduction

Lung cancer is a common cancer and has become the leading cause of deaths from cancer in many developed and developing countries^1^. The majority (approximately 80%) of lung cancer cases are non-small-cell lung cancer (NSCLC) cases, of which 30–50% are adenocarcinoma, the most common histological type^2^. Only 15% of patients with NSCLC adenocarcinoma survive for more than 5 years after primary diagnosis^3^. Cigarette smoking and other noxious particles and gases that favor chronic lung inflammation have been established as risk factors for lung cancer development. Aberrant molecular changes, including oncogene (HER2, BRAF, ROS1 and FGFR1) activation and tumor suppressor genes (GPRC5A, Nkx2-1) inactivation, play important roles in lung cancer development^4–6^. In addition, the tumor microenvironment, consisting of stromal cells, is also an indispensable participant in tumor pathogenesis^7^. Nevertheless, the precise regulatory mechanisms of lung cancer development need to be studied further.

S100A4 (also known as fibroblast-specific protein 1), a member of the S100 calcium-binding protein family, was first cloned in metastatic cells and fibroblasts^8,9^. It is a marker of fibroblasts and a specific subset of inflammatory macrophages^10,11^. S100A4 is expressed in a variety of cells, such as fibroblasts, macrophages, lymphocytes and malignant cells, and plays a crucial role in mediating the interplay between the tumor and stroma^9,12–14^. It is reported to function in both intracellular and extracellular forms. Intracellularly, S100A4 binds to several targets involved in the regulation of angiogenesis, cell survival, motility, invasion or metastasis^15–17^. S100A4 is secreted from both tumor and non-malignant cells and exerts extracellular effects in regulating angiogenesis and cell migration^18,19^. Ablation of S100A4 expression in stromal cells significantly reduces metastatic colonization by regulating the matrix protein tenascin-C and vascular endothelial growth factor-A^13^. We found that S100A4^+^ fibroblasts promoted skin carcinogenesis by maintaining monocyte chemotactic protein-1-mediated macrophage infiltration and chronic inflammation^12^. In addition, using a methylcholanthrene-induced fibrosarcoma model, we found that S100A4^+^ cells prevented carcinoma through collagen production and encapsulation of carcinogens^20^.

Autophagy is a conserved self-cannibalism process in which cellular organelles and proteins are sequestered in autophagosomes and then degraded in bulk in lysosomes, after which cellular compartments are recycled to preserve cellular homeostasis^21,22^. Starvation and other stresses induce autophagy, which clears damaged proteins and organelles and provides energy and building blocks for biosynthesis, crucial for the maintenance of cellular nutrient and energy homeostasis^23^. Dysfunctions in autophagy have been associated with a variety of human diseases, including cancer. Autophagy is a double-edged sword in tumorigenesis, acting both as a tumor suppressor and a protector of cancer cell survival^24^. In epithelial cells, defective autophagy promotes tumor initiation by enhancing oxidative stress and genomic instability as well as activating the transcription factor NRF 2^25^. Defective autophagy also interferes with oncogene-induced senescence and leads to the uncontrolled proliferation of cancer progenitor cells^26^. Conversely, once the malignant phenotype has been established, autophagy serves as a survival mechanism that provides rapidly proliferating cancer cells with nutrients^27^. During autophagy, cytoplasmic LC3 (LC3-I) is enzymatically hydrolyzed and conjugated to the lipid phosphatidyl ethanolamine to form a membrane-type conjugate, LC3-II. Therefore, relative LC3-II level can be used to estimate the extent of autophagy^28^. The generation of autophagosomes can be directly observed under a transmission electron microscope (TEM)^29^. Whether S100A4 can influence tumor development by regulating autophagy is largely unknown.

In this study we showed for the first time that S100A4 plays key roles in lung cancer development by inhibiting autophagy. We found that both extracellular and endogenous S100A4 inhibited starvation-induced autophagy and promoted proliferation of NSCLC cells. Moreover, S100A4 inhibited starvation-induced autophagy and promoted tumor cell proliferation by activating the Wnt/β-catenin pathway in a receptor for advanced glycation end products (RAGE)-dependent manner. Lung tumor growth in S100A4-deficient (S100A4^−/−^) mice was obviously delayed and that expression of autophagy markers in S100A4^−/−^ mice was upregulated. Thus, S100A4 may promote lung tumor development by activating β-catenin signaling and inhibiting autophagy in a RAGE-dependent manner.

## Materials and methods

### Cell lines and mice

Human lung cancer cell line A549 was a kind gift from Dr. Xueqiang Zhao (Tsinghua University School of Medicine). Murine Lewis lung carcinoma cells (LLC) and human lung cancer cells (A549) were cultured in Dulbecco's modified Eagle's medium (Invitrogen, Carlsbad, CA, USA) and RMPI-1640 (Invitrogen, Carlsbad, CA, USA), supplemented with 10% fetal bovine serum and 100 U/ml penicillin–streptomycin (Invitrogen, Carlsbad, CA, USA) at 37 °C in a humidified atmosphere of 5% CO_2_.

C57BL/6 mice were obtained from Vital River (Beijing, China). S100A4 knockout (S100A4^−/−^) mice were purchased from the Jackson Laboratory (Bar Harbor, ME). All mice were bred under specific pathogen-free conditions in the animal facilities at the Institute of Biophysics, Chinese Academy of Sciences. All animal studies were performed with sex- and age-matched mice after being approved by the Institutional Laboratory Animal Care and Use Committee.

### Mouse model of transplanted tumors

Exponentially growing LLC cells were harvested, washed and a suspension of 1 × 10^6^ LLC cells in 100 μl of sterilized phosphate-buffered saline were injected subcutaneously into the abdomen region of the C57BL/6 (control) and S100A4^−/−^ mice. Two days after the injection, the tumor volumes in each mouse were measured. The mice were kept under observation for 20 days and then killed to obtain the tumors. The wet weight of the tumors was balanced for each mouse.

### Tissue microarray immunohistochemistry staining

**Fig. 1:**
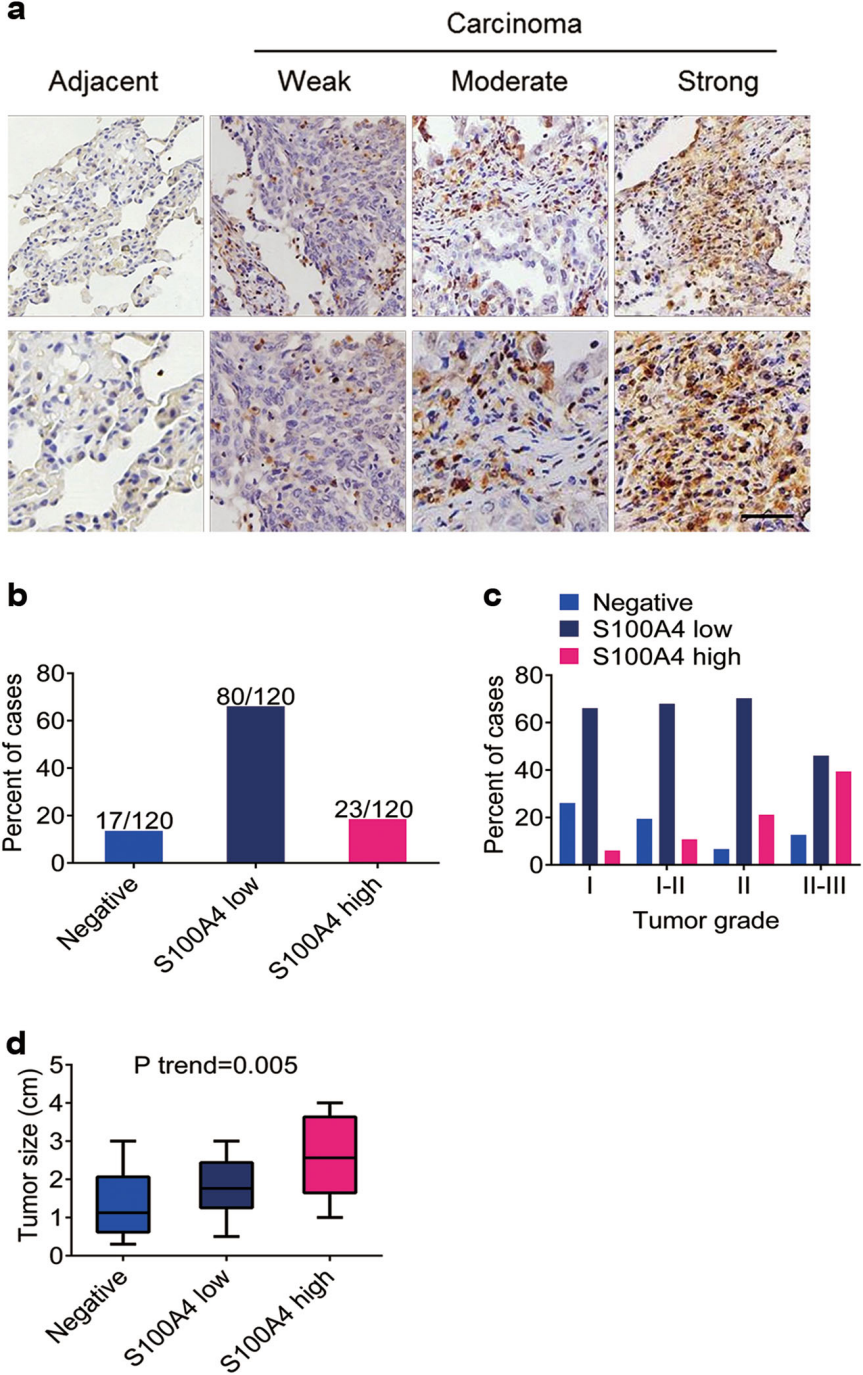
Upregulation of S100A4 expression in NSCLC tissues. Tissue microarray from 120 lung cancer patients was stained with anti-S100A4. **a** Representative S100A4 staining is shown at ×200 (upper) and ×400 magnifications (lower). Scale bar, 50 μm. **b** Percent of the cases expressing S100A4 in carcinoma tissues. **c** Percent of S100A4 negative, low and high expression in different tumor grades. **d** Average tumor size in NSCLC patients with different S100A4 protein levels

Tissue microarrays (TMAs) consisting 120 NSCLC patient cases for immunohistochemistry were purchased from Xin Chao (Shanghai, China). The company provided ethical statement to confirm that the local ethics committees approved their consent procedures and all study participants signed an approved informed consent form. The ethical statement provided by the company and the protocol of experiment had been checked carefully and approved by Ethics Committee of Beijing Jiaotong University. The tissue microarrays were stained with anti-S100A4 (Abcam, Cambridge, UK). In the 120 cases, semi-quantification was carried out for evaluating S100A4 staining according to previous study^30^. Adjacent tissues were also stained as negative control. The intensity of S100A4 expression was scored as follows: 0, negative; 1, weak; 2, moderate; 3, strong (Fig. 1a). The area of positive cancer cells in each microscopic field was categorized as follows: 1, 0 to 25%; 2, 25 to 50%; 3, 50 to 75% or 4, 75 to 100%. The final score was obtained after multiplying the two cores (from 6 to 80). S100A4 negative was defined as a final score from 0 to 5. S100A4 low expression was defined as a final score from 5 to 45. S100A4 high expression was defined as a final score from 45 to 80.

### Immunohistochemistry

A section of each tumor was fixed in 4% paraformaldehyde and then embedded in paraffin for histological hematoxylin–eosin (H&E) (Zhongshanjinqiao, Beijing, China), anti-S100A4 (Abcam, Cambridge, UK) and anti-Ki67 (BD Pharmingen, San Diego, CA) immunohistochemical staining.

For double staining, frozen tumor sections were incubated with anti-S100A4 (Abcam, Cambridge, UK), anti-CD11b (BD Pharmingen, San Diego, CA), anti-F4/80 (BD Pharmingen, San Diego, CA) and anti-α-SMA (Abcam, Cambridge, UK) antibodies, followed by staining with Alexa Fluor 488- or 555-conjugated secondary antibodies (Invitrogen, Grand Island, NY). Sections were evaluated under the microscope (DP71, OLYMPUS) using both bright-field and fluorescence microscopy.

### Reverse transcriptase-polymerase chain reaction (RT-PCR)

Total cellular RNA was extracted from cultured cells using TRIzol reagent (Invitrogen, USA). We synthesized complementary DNA using a Prime Script RT Master Mix Kit (Takara, Tokyo, Japan). Quantitative real-time PCR was performed in duplicate with a SYBR Premix Ex TaqTM Kit (Takara, Japan). The data were analyzed using the 2^−ΔΔCt^ method and normalized against GAPDH expression.

### Western blot

Cultured cells and dissected transplanted tumors were collected; protein was extracted using RIPA lysis buffer (Beyotime, Shanghai, China) supplemented with a cocktail protease inhibitor (Biotool, USA). Based on the protein concentrations measured using a BCA kit (Bio-Rad, Shanghai, China), equal amounts (30 μg) of sample were mixed with 5× SDS loading buffer (Beyotime, China) and ddH_2_O. Samples were heated at 99 °C for 5 min and separated by electrophoresis on a 10% sodium dodecyl sulfate–polyacrylamide gel electrophoresis at 115 V for 1.2 h. Proteins were transferred to a polyvinylidene difluoride membrane at 18 V for 50 min. Membranes were blocked in 5% bovine serum albumin of TBST (Tris-buffered Saline with Tween 20) at room temperature (RT) for 1 h and incubated overnight at 4 °C with the following primary antibodies: Beclin1, LC3-I/II, nuclear factor (NF)-κB p65, phospho-NF-κB p65 (Ser536), RAGE, β-catenin, phospho-β-catenin (Ser552), extracellular signal–regulated kinase-1/2 (ERK1/2), phospho-ERK1/2 (Thr202/Tyr204), Bax and glyceraldehyde 3-phosphate dehydrogenase (GAPDH; 1:1000, Cell Signaling Technology, USA). Membranes were rinsed in TBST, incubated with appropriate secondary antibodies for 1 h at RT and then washed in 1× TBST. After incubation with the ECL plus system (Amersham Biosciences, Sweden), signals were detected using the Image Quant LAS 4000 mini system (GE Healthcare Bio-Sciences, England).

### Analysis of cell viability

For assay of cell viability, cells were grown in a 96-well plate for 5.0 × 10^3^ cells per well. The following day, cells were treated with various chemicals indicated. At 24 h after incubation, cells were measured by using Cell Counting Kit-8 (CCK-8, Dojindo Laboratories, Japan) according to the guidelines from the manufacturer. After being subjected to CCK-8, cells were incubated for 2 h, and then the OD value of cells was measured.

### Transmission electron microscopy

Tissue and cells specimens were processed into approximately 1 mm cubes. Samples were fixed in 2% glutaraldehyde and 2.5% glutaraldehyde post-fixed in 1% osmium tetroxide, rinsed with sodium cacodylate trihydrate buffer, dehydrated with gradient alcohol which was then replaced with propylene oxide and, finally, embedded in Epon 812. After that, semi-thin (1 µm) sections were cut, stained with methylene blue and then viewed under a microscope. Ultra-thin sections were stained with lead citrate and uranyl acetate and examined using a JEM-1400 electron microscope (JEOL, Japan). TEM images of the sections were taken using a camera-specific imaging system for EM (830.10U3 CCD Gatan, USA).

### Recombinant vector transfection and siRNA interference

The S100A4 protein was purchased from Sino Biological company (Catalog: 10185-H01H, Beijing, China). pcDNA3.1 or pcDNA3.1-β-catenin plasmids were obtained from Huiteng Gene (Guangdong, China). pEGFP and pEGFP-S100A4 plasmids were kindly provided by Dr. Yingjie Wu (College of Animal Science and Technology, China Agricultural University, China). Transfection of the vectors was performed using Lipofectamine 2000 according to the manufacturer’s protocols (Invitrogen, 18292-011). S100A4 small interfering RNA (siRNA) and a nonspecific negative control were purchased from Cell Signaling Technology (USA) and transfected using Lipofectamine 2000 according to the manufacturer’s protocols.

### Statistics

All of the data were expressed as the mean ± SEM and analyzed using GraphPad Prism software. Significant differences between mean values were obtained using three independent experiments. Differences between two groups were compared using a two-tailed unpaired Student’s *t*-test analysis. One-way analysis of variance tests with Bonferroni correction were used for multiple comparisons. Correlations between S100A4 expression and clinicopathologic variables were obtained using Spearman’s rank correlation analysis (age, sex, tumor grade and tumor size). *P* < 0.05 was considered statistically significant.

## Results

### The high expression of S100A4 in lung adenocarcinoma tissues is associated with poor prognosis

To identify the relationship between S100A4 expression and lung cancer progression, the expression of S100A4 in lung adenocarcinoma tissues was performed in a TMA by immunohistochemistry. We found that only a few S100A4^+^ cells were detected in paracarcinoma tissues, however, various ranges of immunostaining intensities were observed in lung adenocarcinoma tissues (Fig. 1a). As shown in Fig. 1b, about 14.17% of the lung adenocarcinoma tissues were assessed to be negative expression, and 66.67% and 19.17% of the lung adenocarcinoma tissues were detected with low and high expression of S100A4, respectively. Furthermore, the high expression of S100A4 was significantly correlated with advanced tumor grades (Fig. 1c) and tumor size (Fig. 1d), while there was no significant relation between high S100A4 expression and age factors as well as sex features (Supplementary Table S1). Our results suggest that S100A4 expression may influence tumor size and advanced tumor grades in lung adenocarcinoma cases.

### Extracellular S100A4 suppressed starvation-induced autophagy in both human and mouse lung cancer cells

**Fig. 2:**
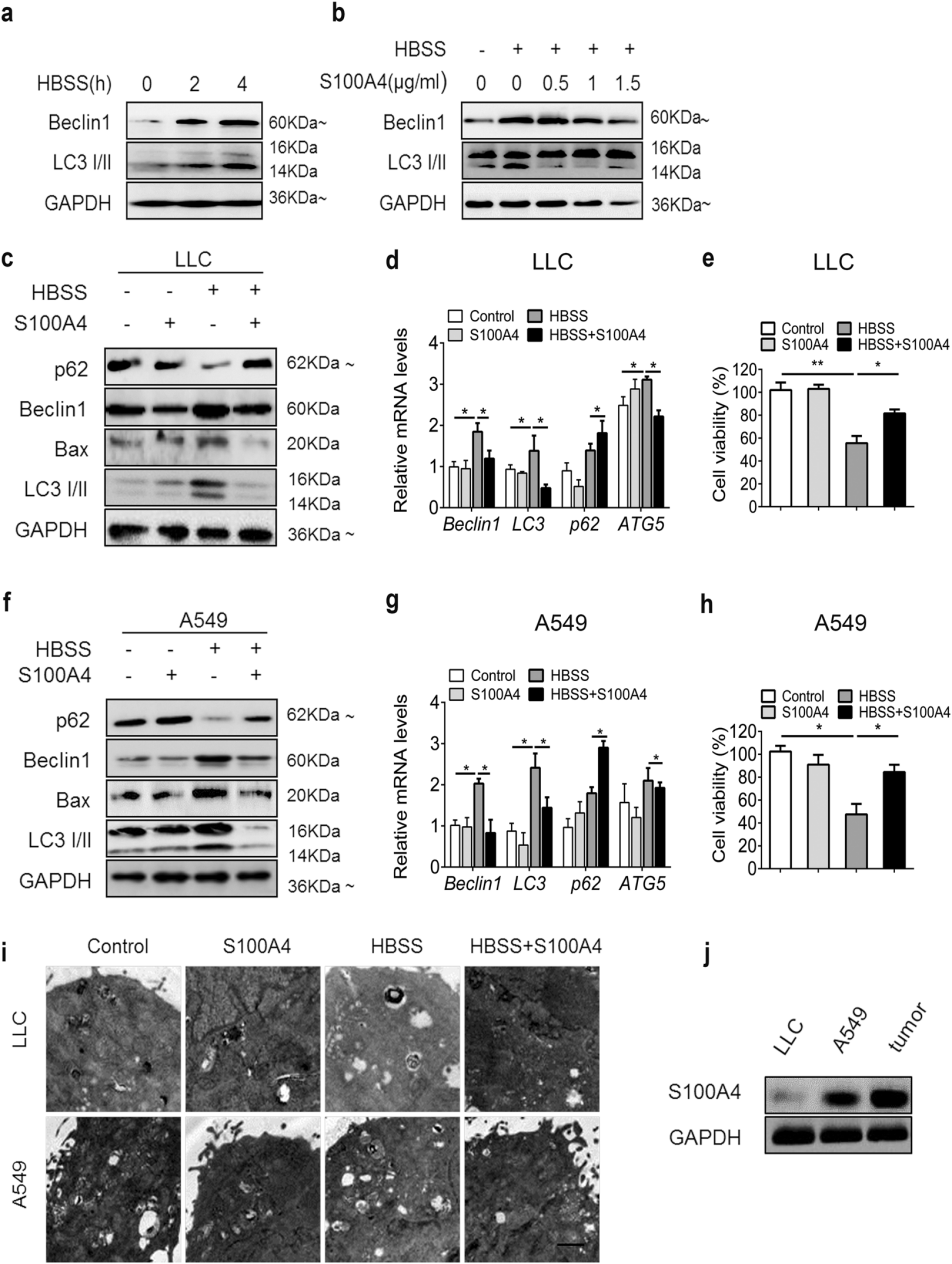
Extracellular S100A4 inhibits autophagy and promotes cell proliferation in LLC and A549 cells. Cells were treated with Hank’s balanced salt solution (HBSS) to induce starvation. **a**, **b** A549 cells were treated with HBSS and S100A4 as indicated. Beclin1 level and LC3-I/II accumulation was determined by western blot analysis. LLC (**c**) and A549 cells (**f**) were treated with or without 1 μg/ml S100A4 in serum-containing medium or HBSS for 4 h. Treated cells were collected and analyzed for p62, Beclin1, Bax, LC3-I/II and GAPDH expression by western blot. Real-time PCR data of the mRNA expression of Beclin1, LC3-I/II, p62 and ATG5 in LLC (**d**) and A549 cells (**g**). Cell viability of LLC (**e**) and A549 cells (**h**) treated with HBSS and S100A4 was measured using a CCK-8 kit. **i** Representative electron microscopy of cells treated with or without 1 μg/ml S100A4 in serum-containing medium or HBSS for 4 h. **j** S100A4 levels in LLC and A549 cells were determined by western blot (tumor section was used as a positive control). Scale bar, 0.25 μm; *p < 0.05, **p < 0.01

Autophagy plays important roles in tumor development^24^. We wondered whether S100A4 affects lung tumor development by regulating autophagy. S100A4 is reported to function both intracellularly and extracellularly^15,17,18^. The secretion of S100A4 by tumor and stromal cells is believed to serve as a key player in promoting the metastasis of cancer cells or affecting angiogenesis^13,31^. To determine whether extracellular S100A4 suppressed autophagy in cancer cells, A549 human lung cancer cells were starved with Hank’s balanced salt solution (HBSS) to induce autophagy and then supplied with S100A4 protein. After treatment with HBSS at the indicated times or with S100A4 protein at the indicated concentrations for 4 h, Beclin1 and LC3-II levels increased most significantly after 4 h in HBSS (Fig. 2a) and decreased most significantly with S100A4 protein treatment (1 μg/ml) in the A549 cell line (Fig. 2b). Then, the expressions of LC3-II and Beclin1 were further examined in LLC and A549 cell lines after 1 μg/ml of S100A4 protein treatment for 4 h. Western blot analysis showed that after starved with HBSS, protein levels of Beclin1, Bax and LC3-II were greatly reduced, while the level of p62 was significantly elevated when S100A4 protein was supplied to LLC cells (Fig. 2c) and A549 cells (Fig. 2f). Similar results were obtained using real-time RT-PCR (Fig. 2d, g) and TEM (Fig. 2i). In addition, S100A4 led to significantly increased cell viability after cells were subjected to HBSS (Fig. 2e, h). We also found that the expression of S100A4 was relatively low in LLC cells when compared with A549 cells and tumor sections (Fig. 2j). These results strongly suggest that exogenous S100A4 is an inhibitor of autophagy and promotes cell proliferation in NSCLC cells.

### Overexpression of S100A4 inhibited starvation-induced autophagy, whereas siRNA-mediated suppression of S100A4 increased autophagy in A549 cells

**Fig. 3:**
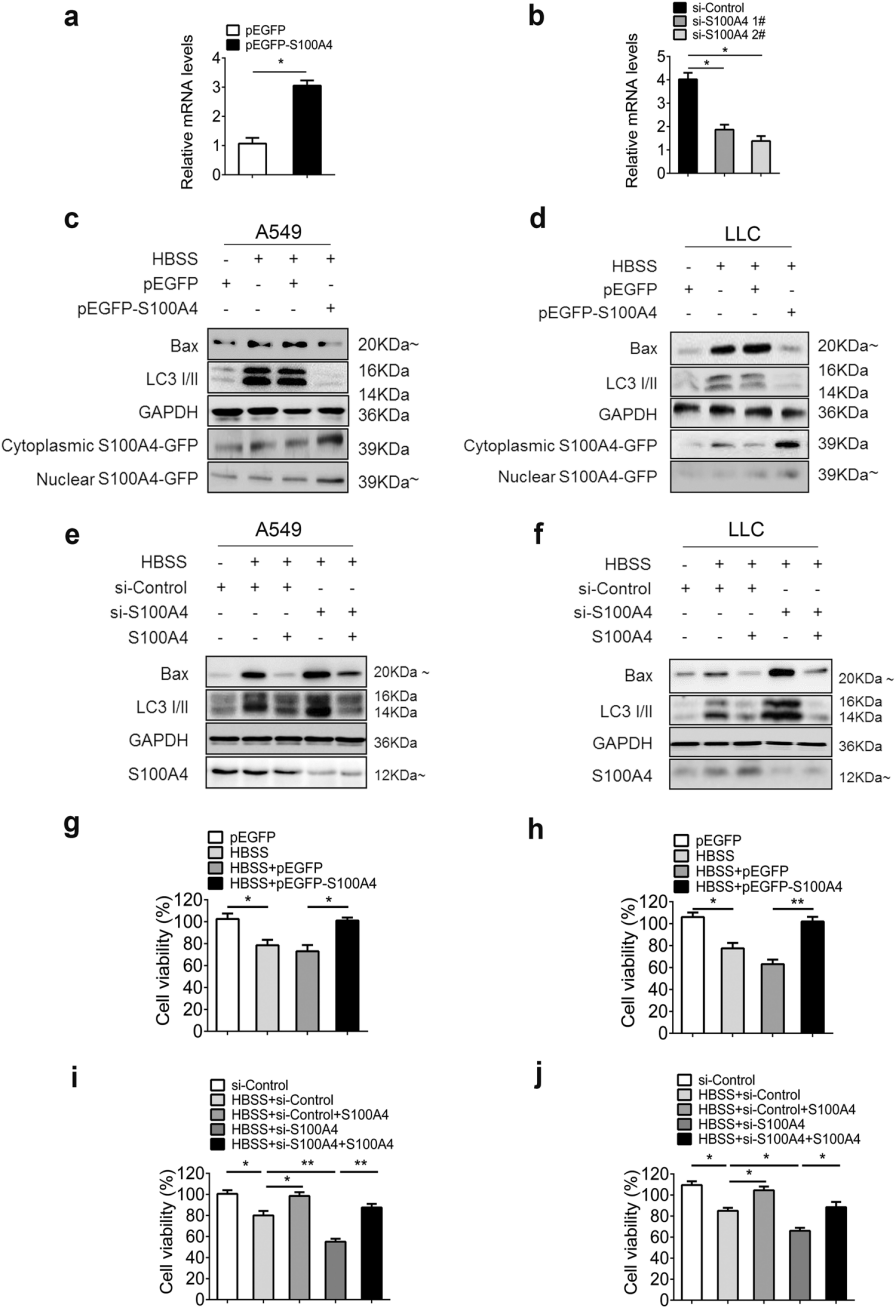
In A549 and LLC cells, overexpression of S100A4 inhibited autophagy whereas siRNA-mediated suppression of S100A4 increased autophagy. **a** A549 cells were transfected with either a plasmid overexpressing S100A4 (pEGFP-S100A4) or a control plasmid (pEGFP). At 48 h after transfection, the cells were incubated with HBSS instead of RMPI-1640 for 4 h; mRNA expression of S100A4 was analyzed by real-time PCR. **b** A549 cells were exposed to S100A4 siRNA or control siRNA for 24 h, then HBSS instead of RMPI-1640 was applied in cell lines for 4 h; S100A4 mRNA expression was detected by real-time PCR. Cell lysates from A549 (**c**) and LLC (**d**) overexpressing S100A4 were subjected to western blot analysis with the indicated antibodies. A549 (**e**) and LLC (**f**) cells treated with S100A4 siRNA were collected and analyzed with the indicated antibodies. The cell viability of A549 (**g**) and LLC cells (**h**) was assessed by CCK-8 assay after exposure to pEGFP-S100A4, respectively. The effect of S100A4 siRNA on cell viability of A549 (**i**) and LLC cell line (**j**) was also assessed; *p < 0.05, **p < 0.01

We then investigated whether autophagy was inhibited by endogenous S100A4. As shown in Fig. 3a, the expression of S100A4 messenger RNA (mRNA) was upregulated when A549 cells were transfected with a plasmid overexpressing S100A4 (pEGFP-S100A4). The knockdown of S100A4 in A549 cells was confirmed by RT-PCR (Fig. 3b). The expression of S100A4 in purified cell fractions was also confirmed by the following western blot. Western blot data showed an enhanced inhibition of Beclin, Bax and LC3-II expression as well as elevated p62 levels in A549 cells overexpressing S100A4 (Fig. 3c), and similar results were obtained from LLC cells (Fig. 3d). Meanwhile, the effects of S100A4-siRNA on autophagy were also assessed. Silencing of S100A4 consistently led to an increased Beclin1, Bax and LC3 conversion and reduced p62 level, which was reversed by S100A4 treatment to lung cancer cells once again (Fig. 3e, f). Overexpression of S100A4 significantly increased cell viability (Fig. 3g, h) while knockdown of S100A4 reduced cellular viability in HBSS-treated cells (Fig. 3i, j). These data demonstrate that endogenous S100A4 promotes cell proliferation and has inhibitory effects towards starvation-induced autophagy.

### S100A4 inhibited autophagy and promoted A549 cells proliferation via the Wnt/β-catenin pathway

**Fig. 4:**
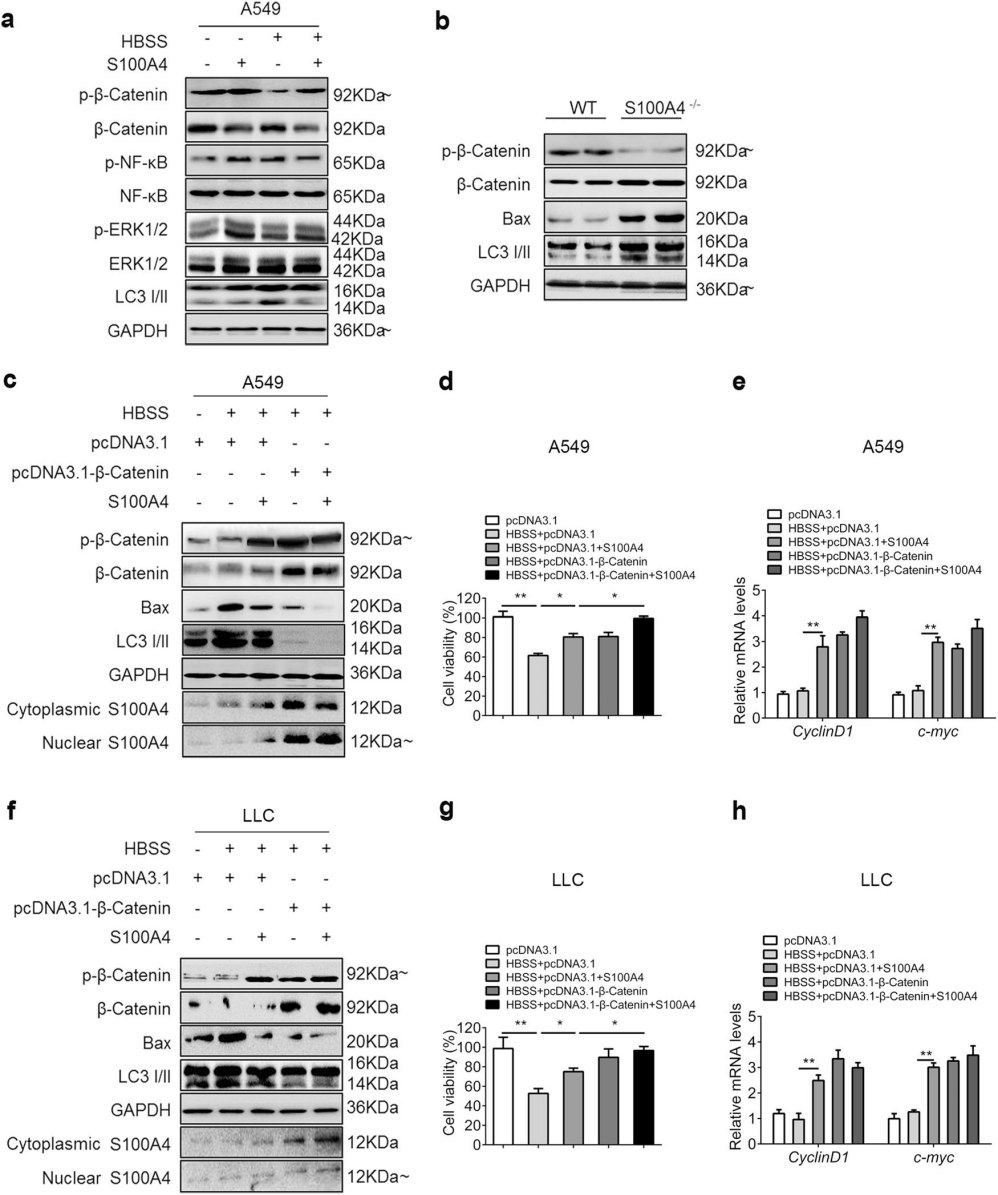
S100A4 inhibited autophagy to promote tumor cell survival through β-catenin signaling. **a** A549 cells were treated with HBSS and S100A4 as indicated. The indicated signal pathways were analyzed by western blot in A549 cells. **b** Western blot analysis of β-catenin, phospho-β-catenin, Bax, LC3-I/II and GAPDH protein levels in tumors from S100A4^−/−^ and WT mice. A549 (**c**) and LLC (**f**) cells were transfected with plasmid overexpressing β-catenin (pcDNA3.1-β-catenin) or control plasmid (pcDNA3.1) and then treated with 1 μg/ml S100A4 in HBSS for 4 h. At 48 h after transfection, cell lysates were subjected to western blot analysis with the indicated antibodies. Cell viability of A549 cells (**d**) and LLC cells (**g**) treated with pcDNA3.1-β-catenin was measured using a CCK-8 kit. mRNA levels of target genes (*CyclinD1* and *c-myc*) in β-catenin pathway from A549 (**e**) and LLC (**h**) cells was measured by RT-PCR; *p < 0.05, **p < 0.01

We further tested the signaling pathways that S100A4 might be involved in during autophagy using the A549 cell line. A549 cells were cultured with HBSS and S100A4 to analyze the expression of pathway-related proteins. After treatment with S100A4 for 4 h, we found that both the overall protein and phosphorylation level of P65 and ERK1/2 were unaffected in A549 cells. However, supplementation of S100A4 led to a marked upregulation in phosphorylated β-catenin (Ser552) levels accompanied with the impaired Bax and LC3-II expression (Fig. 4a). The Wnt/β-catenin pathway of tumor tissues was also analyzed, and similar results were found. As shown in Fig. 4b, S100A4 deficiency resulted in a marked decrease in phosphorylated β-catenin levels in tumors from S100A4^−/−^ mice and increased Bax and LC3-II levels.

The effects of overexpression of β-catenin on the expression levels of phospho-β-catenin and LC3 conversion were further studied. We confirmed that β-catenin was overexpressed and found that both the cytoplasmic and nuclear expression of S100A4 were also upregulated following overexpression of β-catenin in lung cancer cells by western blot (Fig. 4c, f). Upregulation of β-catenin consistently led to a decrease in LC3 conversion as well as Bax level, differing from the control, which failed to show any effects on the steady-state levels of LC3 conversion (Fig. 4c, f). Figure 4d, g demonstrated that overexpressed β–catenin contributed to cellular proliferation in lung cancer cells. The transcriptional activity of β–catenin were further studied by monitoring the mRNA levels of *CyclinD1* and *c-myc*, which indicated that S100A4 promoted the transcription activity of wnt/β–catenin pathway (Fig. 4e, h).

**Fig. 5:**
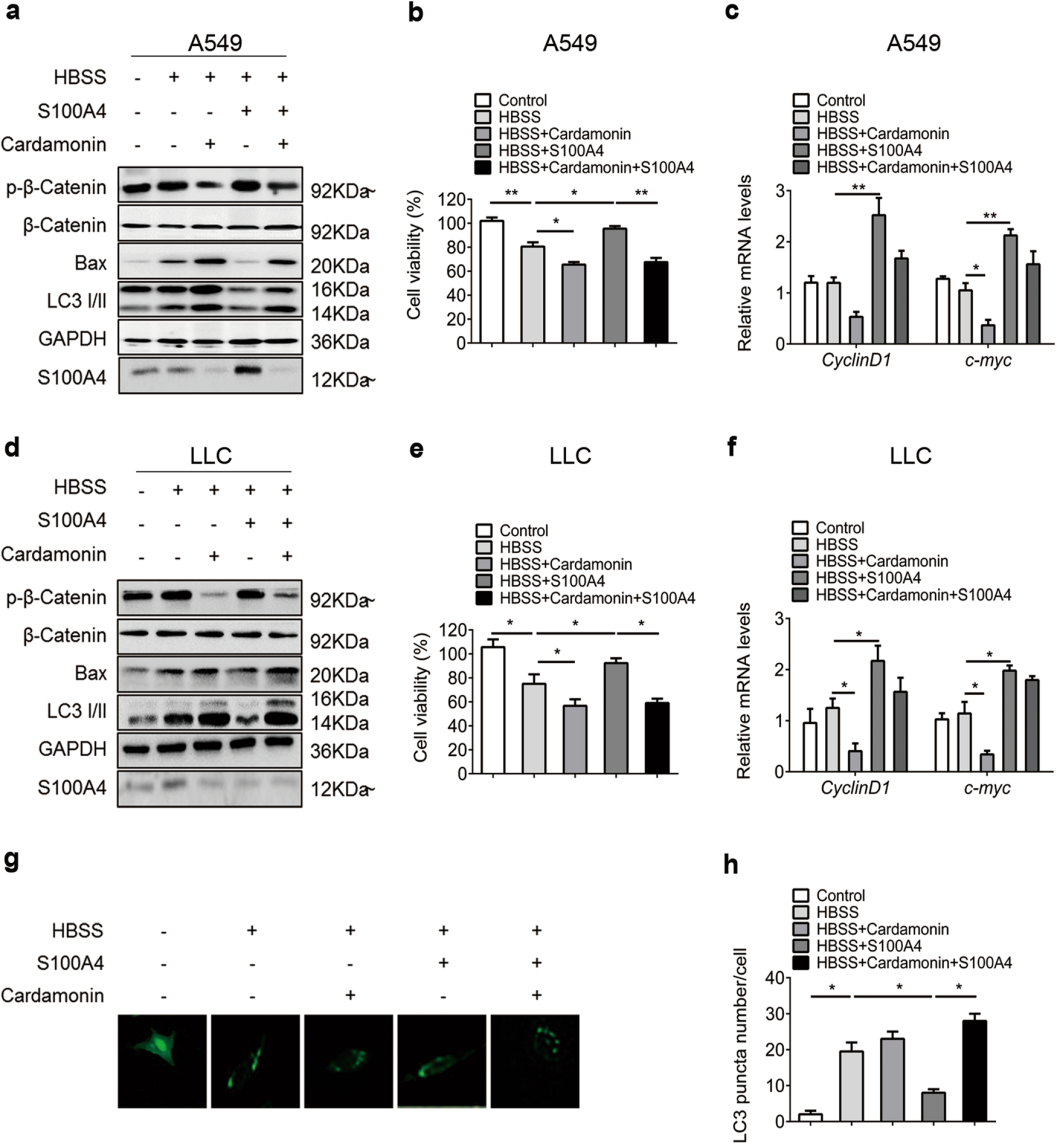
β-catenin inhibitor in A549 cells abolished the effects of S100A4 on autophagy. A549 and LLC cells were cultured with the β-catenin inhibitor cardamonin (20 μmol/ml) for 1 h, then cells were stimulated with or without S100A4 (1 μg/ml) in HBSS for 4 h. The treated A549 cells (**a**) and LLC cells (**d**) were collected and analyzed for β-catenin, phospho-β-catenin, Bax and LC3-I/II protein expression. Cell viability of A549 (**b**) and LLC (**e**) cells treated with cardamonin was detected by a CCK-8 kit. mRNA levels of target genes (*CyclinD1* and *c-myc*) in β-catenin pathway from A549 (**c**) and LLC (**f**) cells was measured by RT-PCR. **g** A549 cells were transfected with GFP-LC3-I/II plasmid (1 ng/μl) for 6 h. **h** Representative images of GFP-LC3 staining and quantification of LC3 puncta numbers per cell in cells are shown; *p < 0.05, **p < 0.01

Cardamonin is a chalcone from *Aplinia katsumadai Hayata* that inhibits intracellular β-catenin levels^32^. To further confirm whether activated β-catenin signaling was necessary for S100A4-inhibited autophagy, A549 and LLC cells were treated with cardamonin, and the effects of S100A4 on autophagy were determined by evaluating LC3-II and Beclin1 abundance. In cells treated with cardamonin, supplementation with S100A4 protein led to abolished β-catenin levels and relatively steady Bax and LC3-II expression (Fig. 5a, d). We also found the S100A4 level was negatively influenced by cardamonin in this process. As shown in Fig. 5b, e, when A549 and LLC cells were treated with cardamonin, S100A4 failed to increase the cell viability. The decreased transcription of β-catenin pathway target genes by cardamonin was also confirmed in the two cell lines (Fig. 5c, f). Induction of autophagy was also detected by quantification of LC3 puncta in cells, where the β-catenin signaling was evaluated as inhibited cells versus neighboring cells where β-catenin signaling was uninhibited. The majority of cells with inhibited β-catenin had significantly more LC3 puncta than those without inhibited β-catenin (Fig. 5g, h).These results suggest that S100A4 inhibited autophagy to promote tumor cell survival via the Wnt/β-catenin pathway.

### S100A4-induced β-catenin signaling to inhibit autophagy in A549 cells is RAGE dependent

**Fig. 6:**
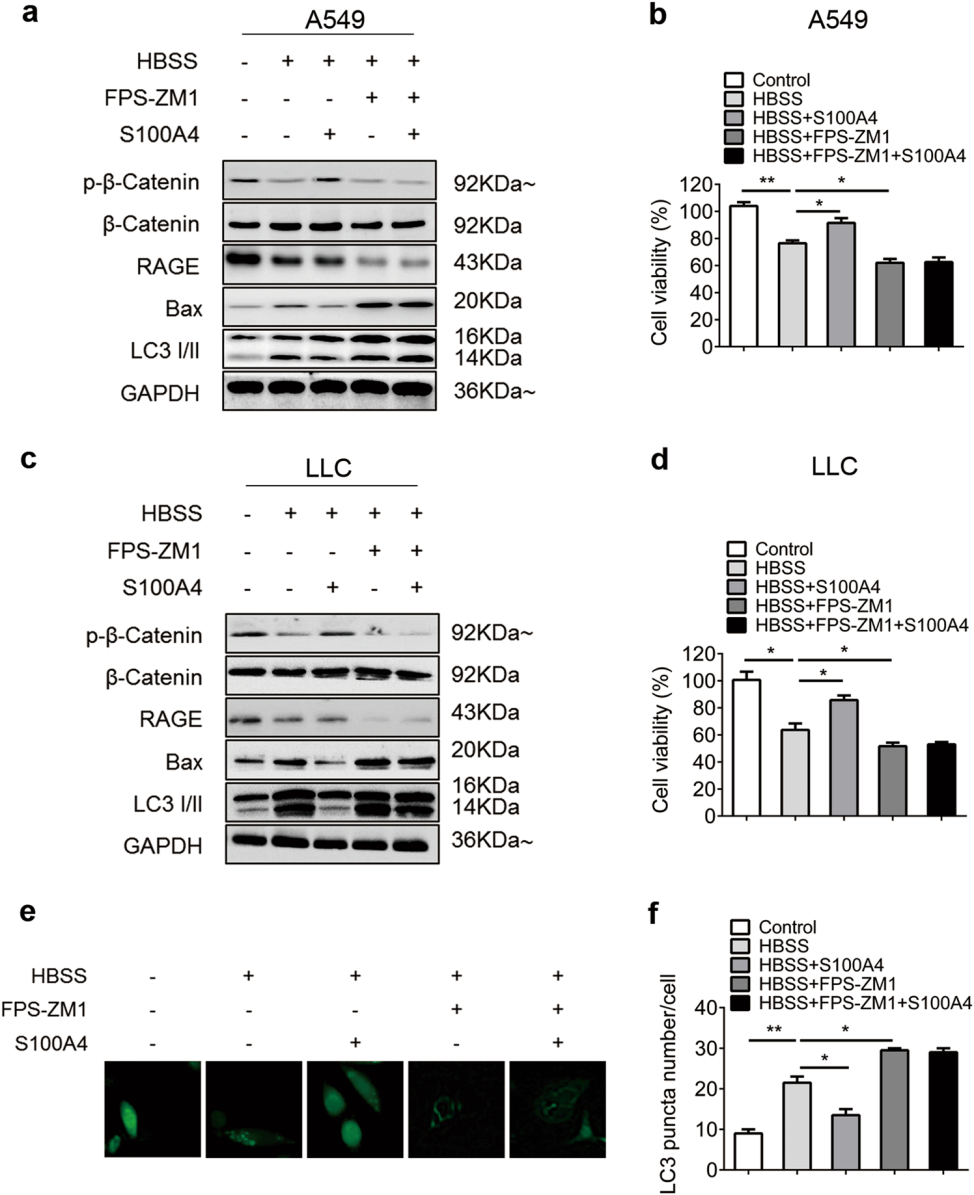
S100A4-induced autophagy in A549 cells is RAGE dependent. A549 and LLC cells were incubated with the RAGE-specific blocker FPS-ZM1 (75 nmol/μl) for 30 min before being treated with S100A4 (1 μg/ml) in HBSS for 4 h. The treated A549 cells (**a**) and LLC cells (**c**) were collected and analyzed for the expression of β-catenin, phospho-β-catenin, RAGE, Bax and LC3-I/II by western blot. Cell viability of A549 (**b**) and LLC (**d**) cells treated with FPS-ZM1 was determined by a CCK-8 kit. **e** A549 cells were transfected with GFP-LC3-I/II plasmid (1 ng/μl) for 6 h. **f** Representative images of GFP-LC3 staining in cells and quantification of LC3 puncta numbers per cell are shown; *p < 0.05, **p < 0.01

RAGE is a well-accepted interaction partner for S100A4. We analyzed whether the S100A4-induced β-catenin signaling in A549 cells was RAGE dependent. The RAGE-specific inhibitor FPS-ZM1 was administered to A549 cells. RAGE inhibition in A549 and LLC cells both resulted in the abolishment of the inhibitory effects of S100A4 on autophagy (Fig. 6a, c). As shown in Fig. 6b, d, when starved cells were exposed to FPS-ZM1, the cell viability was not affected by S100A4. Induction of autophagy was also determined by quantification of LC3 puncta in RAGE-inhibited cells and their controls. The majority of cells with RAGE inhibition had significantly more LC3 puncta than those without RAGE inhibition (Fig. 6e, f).These results indicate that S100A4 inhibited autophagy in a RAGE-dependent manner.

### S100A4 promoted lung tumor development by inhibiting autophagy

**Fig. 7:**
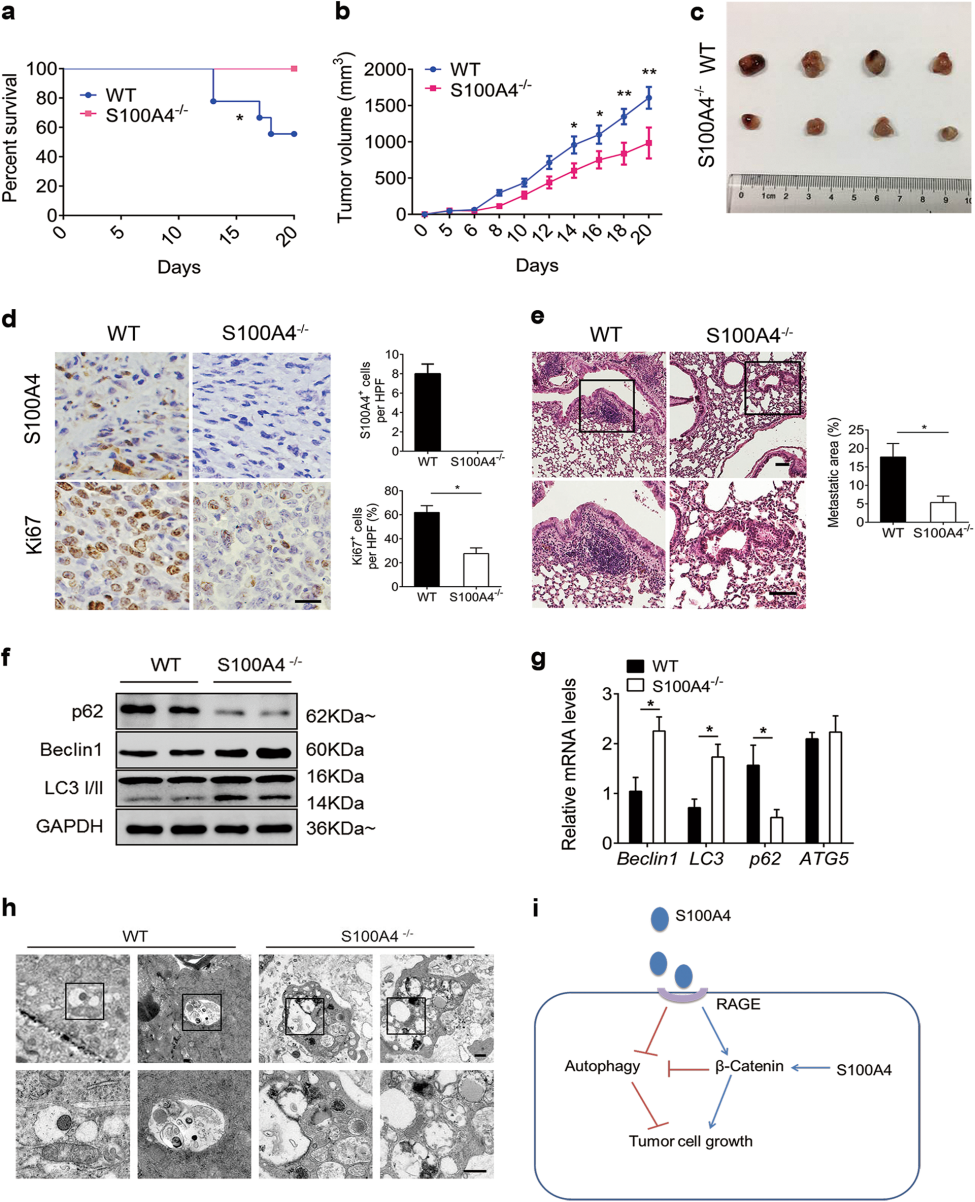
S100A4 promoted lung tumor development by inhibiting autophagy. Groups of C57BL/6 mice and S100A4^−/−^ mice (*n* = 9 per group) were subcutaneously injected with 1 × 10^6^ LLC cells. **a** The survival analysis of lung tumor-bearing mice. **b** Tumor volumes were monitored every other day. Representative data from three independent experiments are shown. **c** On day 20 after tumor inoculation, mice were killed and tumors from the two groups are shown. **d** The tumor sections were stained for S100A4 and Ki67. Scale bar, 50 μm. HPF high power field. **e** Representative H&E-stained lung sections of WT and S100A4^−/−^ mice. The percentage of the metastatic area was quantified using Image-Pro Plus (IPP). **f** The levels of the autophagy-related proteins p62, Beclin1 and LC3-I/II in tumors from WT and S100A4^−/−^ mice were detected by western blot. **g** mRNA expression of Beclin1, LC3-I/II, p62 and ATG5 in tumors from S100A4^−/−^ and WT mice was detected by real-time PCR. **h** Representative electron microscopy of dissected tumors from S100A4^−/−^ and WT mice. Scale bar, 0.25 μm. **i** Schematic of the S100A4 acting model in lung cancer. S100A4 is a negative regulator of autophagy via activation of the β-catenin signaling pathway and this process is RAGE dependent. The autophagy inhibitor S100A4 contributed to lung tumor cell growth; *p < 0.05, **p < 0.01

To further investigate the role of S100A4 in lung tumor development, LLC cells were implanted subcutaneously in S100A4^−/−^ mice and wild-type (WT) control mice. Tumor growth was monitored, and tumor volume was measured every other day. As shown in Fig. 7a, the survival rate of S100A4^−/−^ mice bearing LLC tumors was significantly increased as compared with WT mice. The tumors in the WT mice grew rapidly, whereas tumor development was severely impaired in the S100A4^−/−^ mice. On day 14, WT mice had a mean tumor volume of 957.26 mm^3^ compared with 602.84 mm^3^ for the S100A4^−/−^ mice (Fig. 7b). At the end of the experiment, the tumor volumes were 1607.93 mm^3^ for the WT group and 984.71 mm^3^ for the S100A4^−/−^ group (Fig. 7b, c). Tumor sections were then stained with immunohistochemistry as shown in Fig. 7d. A large number of S100A4^+^ cells were identified in the tumors from WT mice and none were detected in the tumors from S100A4^−/−^ mice. Using double staining, we found that most of the S100A4^+^ cells were fibroblasts, i.e., 69.31% of the S100A4^+^ cells were α-SMA^+^. The S100A4^+^ cells rarely expressed Gr-1, CD4 or CD8, and only a small number of S100A4^+^ cells were positive for F4/80^+^ (Supplementary Figure S1). Moreover, the percentage of Ki67^+^ (proliferating) cells in tumors was decreased significantly in S100A4^−/−^ mice compared with WT mice (61.89% vs 27.64%) (Fig. 7d). In addition, the percentage of metastatic colonization in the lungs was also obviously reduced in the S100A4^−/−^ group compared with the WT group (5.34% vs 17.69%, Fig. 7e). Furthermore, autophagy markers were detected in tumor tissue sections from S100A4^−/−^ and WT mice. p62, LC3-II and Beclin1 protein expression were analyzed using western blots. As shown in Fig. 7f, autophagy levels increased significantly in tumors from S100A4^−/−^ mice compared with WT mice (Fig. 7f). S100A4 deficiency resulted in elevated mRNA expression of Beclin1, LC3 and decreased mRNA expression of p62, a gene which was negatively correlated with autophagy level (Fig. 7g). TEM further confirmed that the number of autophagosomes and autolysosomes obviously increased in tumors from S100A4^−/−^ mice (Fig. 7h). These results indicate that S100A4 promoted lung tumor development by inhibiting autophagy.

## Discussion

In this study, we found that upregulation of S100A4 expression in lung adenocarcinoma tissues is closely related to advanced tumor grades. Furthermore, in vitro, S100A4 activated β-catenin signaling in lung cancer cells and increased tumor cell viability via inhibiting autophagy in a RAGE-dependent manner. S100A4 deficiency promoted autophagy and hindered lung tumor growth in vivo. Our results demonstrated that S100A4 promoted lung tumor development by upregulating the β-catenin pathway and inhibiting autophagy.

S100A4 plays important roles in cancer progression and metastasis in different ways^17^. It promotes tumor growth and progression mainly by stimulating cell survival and proliferation, increasing motility and invasion of tumor cells, modifying adhesion of tumor cells, maintaining chronic inflammation and promoting angiogenesis^9,12,13,31^. S100A4 also participates in cell apoptosis by interacting with tumor suppressors^33^. Downregulation of S100A4 enhances the sensitivity of human lung cancer cells to radiotherapy^34^. S100A4 can be one of the prognostic biomarkers in lung cancer^35^. It has been reported that elevated S100A4 expression was highly correlated with poor prognosis in lung adenocarcinoma, lung squamous cell carcinoma and other subtypes of non-small celllung carcinomas^35–38^. However, whether S100A4 can promote tumor development by regulating autophagy has not been determined. Our results demonstrated that S100A4 expression in tumor tissues from lung adenocarcinoma patients is closely linked to tumor progression patient-derived tissues and this study is the first report in which S100A4 is shown to promote lung tumor growth by downregulating autophagy.

Autophagy, a cellular waste disposal process, has well-established tumor-suppressive properties^39^. Under metabolic stress, autophagy is a survival strategy in cancer cells. Although autophagy helps cancer cells address the increased demand for nutrients caused by cell growth and proliferation, there is more evidence that tumor suppressor genes are associated with the activation of autophagy, and the enhancement of autophagy often leads to cell death^40^. Autophagy inhibits breast cancer stem-like cells by suppressing the Wnt/β-catenin signaling pathway^41^. A defect in autophagy was sufficient to promote tumorigenesis by altering NF-κB regulation^42^. Furthermore, autophagy gene-deficient mice show high incidences of primary lung cancer^43^, which is consistent with our results that show that impaired autophagy induced rapid cell proliferation in implanted tumors. Excessive autophagy is closely linked to the low survival rate of lung cancer in lung cancer patients after surgery^44^. Here, we found that both extracellular treatment and intracellular overexpression of S100A4 suppressed autophagy and increased cell viability in lung cancer cells. Moreover, silencing S100A4 in lung cancer cells significantly increased autophagy and inhibited tumor cell proliferation. These results suggest that intracellular expressed S100A4 and extracellular S100A4 secreted by stromal cells can accelerate cell proliferation through suppressing autophagy. Our results demonstrate that S100A4 promoted tumor progression by affecting autophagy, which might be used as a therapeutic target in the development of future lung cancer therapies.

The Wnt/β-catenin pathway maintains normal tissue homeostasis and is aberrantly activated in the majority of tumors^45,46^. β-Catenin is an essential transcriptional co-regulator in the canonical Wnt pathway, forming complexes with the T cell-specific transcription factor/lymphoid enhancer-binding factor 1 (TCF/LEF) family of transcription factors to control target gene expression^47^. Mutations of Wnt pathway components prevent the destruction of β-catenin, leading to increased TCF-regulated transcription of Wnt/β-catenin target genes that promote cell proliferation and facilitate tumorigenesis^48–50^. In this study, we found that S100A4 upregulated β-catenin expression and inhibited autophagy. The β-catenin signaling pathway regulates cell apoptosis and autophagy^51,52^. Inhibition of β-catenin significantly increased LC3-II expression and induced autophagic cell death^53^. Conversely, the upregulation of β-catenin signaling reduces Beclin1 expression and rescues endothelial cells from endostatin-induced autophagy^54^. Consistent with these reports, we showed that activated β-catenin signaling resulted in impaired autophagy which was induced by starvation and promoted proliferation in lung cancer cells, and cell autophagy was reversed by the overexpression of β-catenin, indicating the critical role of β-catenin in S100A4-induced autophagy inhibition. Furthermore, inhibition of RAGE, one of the S100A4 receptors, abolished the effect of S100A4 on autophagy and cell proliferation. However, the detailed mechanisms by which S100A4 interacts with autophagy in tumor development still need further investigation.

In conclusion, our results demonstrate that both extracellular and intracellular S100A4 promoted lung tumor development by activating the β-catenin pathway and inhibiting RAGE-dependent autophagy (Fig. 7i). The results provide direct evidence of cross talk between S100A4, autophagy and tumor growth and reveal a novel mechanism for lung tumor development. The S100A4-related microenvironment may provide a promising clinical strategy for lung cancer prevention and treatment.

## Electronic supplementary material

Supplementary information
