# Supplementary material for: S100A4 promotes lung tumor development through β-catenin pathway-mediated autophagy inhibition
Source: Cell Death Dis. 2018 Feb 15;9(3):277. doi: 10.1038/s41419-018-0319-1 (PMC5833421; doi:10.1038/s41419-018-0319-1)
Supplement: Supplementary file 1 — Supplementary information [file 41419_2018_319_MOESM1_ESM.docx]

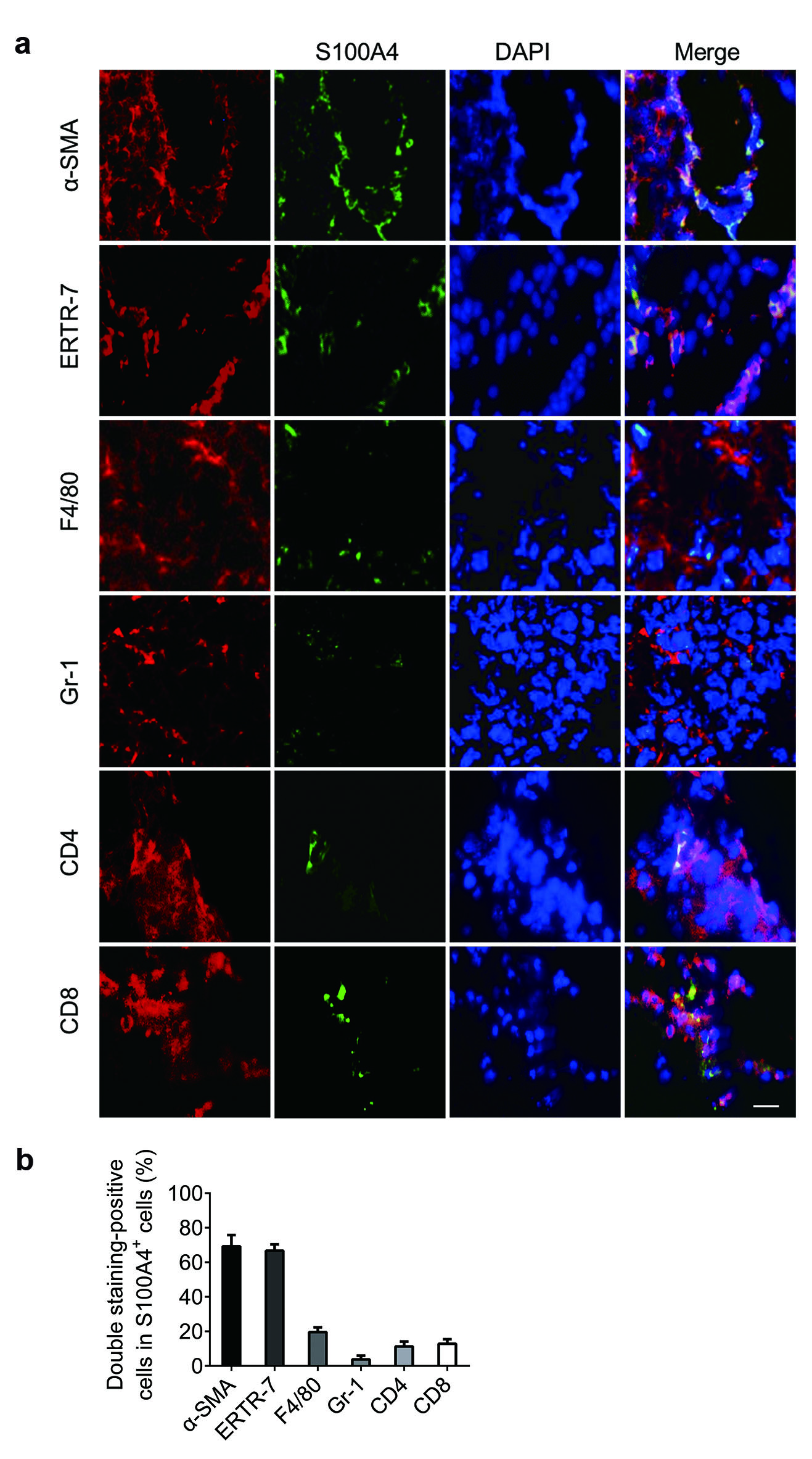


**Supplementary Figure S1.** The majority of S100A4^+^ cells that accumulated in transplanted tumors were fibroblasts. Group of C57BL/6 mice (n=9) were subcutaneously injected with 1×10^6^ LLC cells. On day 20 after tumor inoculation, mice were sacrificed and tumor sections were prepared. (A) Double immunohistochemical staining of S100A4 (green) with α-smooth muscle actin (α-SMA), ERTR-7, F4/80, Gr-1, CD4 and CD8 (red) in the tumor tissue. Nuclei were counter-stained with DAPI (blue). Scale bar, 50μm. (B) Percentages of double staining-positive cells in S100A4^+^ cells were shown.

**Supplementary Table S1.** The association of S100A4 expression with clinical features in lung adenocarcinoma patients.

|  |  |  | |  |
| --- | --- | --- | --- | --- |
|  |  | S100A4 expression | |  |
| Characteristics | Negative  (N=17) | Low  (N=80) | High  (N=23) | P value |
| Age, N (%) |  |  |  |  |
| ≥62 | 7 (41.18) | 42 (52.50) | 14 (60.87) | 0.226 |
| <62 | 10 (58.82) | 38 (47.50) | 9 (39.13) |  |
| Sex |  |  |  |  |
| Male | 4 (23.53) | 33 (41.25) | 10 (43.48) | 0.247 |
| Female | 13 (76.47) | 47 (58.75) | 13 (56.52) |  |
| Tumor grade |  |  |  |  |
| I | 4 (23.53) | 10 (12.50) | 1(4.35) | **0.003** |
| I-II | 7 (41.18) | 24 (30.00) | 4 (17.39) |  |
| II | 4 (23.53) | 39 (48.75) | 12 (52.17) |  |
| II-III | 2 (11.76) | 7 (8.75) | 6 (26.09) |  |
| Tumor size(cm) |  |  |  |  |
| - | 1.34(range:0.5-6) | 1.50(range:0.5-6) | 2.50(range:1-4) | **0.005** |

P for trend, bold for statistically significant.
